# Climate's silent impact: reframing primary care in the era of environmental distress

**DOI:** 10.3389/fmed.2024.1384786

**Published:** 2024-05-15

**Authors:** Waseem Jerjes

**Affiliations:** ^1^Research and Development Unit, Hammersmith and Fulham Primary Care Network, London, United Kingdom; ^2^Faculty of Medicine, Imperial College London, London, United Kingdom

**Keywords:** healthcare, mental health, environmental, climate change, natural disaster

## Introduction

In the evolving healthcare narrative, the pronounced relationship between environmental shifts and human health reflects the global impacts of climate change. These include rising temperatures, erratic weather patterns, and increasing natural disasters, affecting not only our physical environment but also our collective mental wellbeing. The WHO predicts that between 2030 and 2050, climate change will result in ~250,000 additional deaths per year, with significant implications for mental health (1).

## Climate change and mental health: a growing concern

The effects of these environmental disruptions ripple through communities, inducing anxiety, stress, and trauma (2). Now, more than ever, this connection of climate change and mental health is demanding attention. The significance lies not just in acknowledging this connection but in understanding its multifaceted implications, especially within primary care settings.

Simultaneously, the subtle, long-term changes brought about by global warming also weigh heavily on mental wellbeing (2). Watching landscapes alter, hometowns become uninhabitable, and traditional ways of life dissolve can foster solastalgia, the emotional distress caused by environmental change in one's home environment (3–5).

Growing up amidst dramatic climate shifts, children and adolescents face unique psychological challenges, including early-onset anxiety and a pervasive sense of hopelessness (6, 7). Furthermore, these chronic stresses potentially exacerbate pre-existing mental health conditions and create a cascade of challenges that could persist into adulthood.

The deepening crisis of climate change permeates numerous aspects of our lives (6). Yet, its complex relationship with mental health remains inadequately explored, particularly within primary care setting. The multifaceted effects of climate change, from direct physical impacts such as droughts and floods to the subtler, indirect consequences like diminished community cohesion due to forced relocations, exacerbate a spectrum of mental health challenges. These span from episodic distress and anxiety to long-term conditions like depression and PTSD (8, 9).

## Challenges and opportunities in primary care

At the frontline of healthcare delivery, primary care is crucial in understanding and addressing the barriers to integrating mental health care amidst climate change. These barriers include lack of training, resource constraints, and insufficient policy support. Conversely, facilitators such as interdisciplinary collaboration, robust policy frameworks, and community engagement can significantly enhance this integration (10).

The significance of clearly identifying the key stakeholders crucial for effectively implementing these recommendations is universally acknowledged. As such, this opinion paper is directed not only at primary care practitioners but also at primary care networks, professional associations, health ministries, and policymakers. Each entity plays a vital and distinct role in embedding considerations of climate change into mental health care (11).

The primary care setting, with its emphasis on holistic, patient-centered care, is uniquely positioned to detect, address, and support those struggling with climate-related psychological distress (12, 13). Physicians, nurse practitioners, and healthcare workers at this level often have an ongoing relationship with patients, allowing for the detection of subtle behavioral shifts and the provision of timely interventions.

However, achieving this requires integrating a new dimension into primary care, an awareness and understanding of the environmental context. Just as a primary care physician might inquire about personal and occupational stresses, the discussion must expand to include environmental anxieties and exposures. Regular screenings, not just for traditional ailments but also for signs of environmental distress, can aid in early detection and management (14). Additionally, given the community-centered nature of primary care, physicians can also play an instrumental role in fostering community resilience, advocating for community-based support systems and interventions that cater to collective traumas and anxieties.

Integrating climate change-related mental health care within primary care settings presents unique challenges and opportunities. The barriers include the current lack of specific training for primary care providers in addressing environmental impacts on mental health, the limited availability of mental health resources, and the overarching need for more supportive healthcare policies (15). However, facilitators are also emerging, such as increased awareness of climate change impacts, growing advocacy for mental health services, and the development of new interdisciplinary training programs that include environmental health. Addressing these factors effectively requires a coordinated effort from healthcare providers, policymakers, and the community at large (Table 1).

**Table 1:** Strategic framework for integrating climate resilience into mental health care in primary care settings.

**Aspect**	**Challenges**	**Opportunities**	**Strategic actions**
Training and education	Lack of specific training on climate impacts on mental health.	Development of interdisciplinary programs including environmental health.	Develop specialized training modules on climate-related mental health for primary care practitioners.
Resource availability	Limited mental health resources.	Increased funding for climate-related mental health interventions.	Advocate for increased funding and create partnerships for resource development.
Policy support	Insufficient policies for integration of mental health and climate resilience.	Robust frameworks promoting mental health services integration into primary care.	Collaborate with health ministries to develop supportive policies and frameworks.
Community engagement	Diminished cohesion due to relocations and disasters.	Primary care settings fostering community resilience through support systems.	Initiate community-based programs and support networks to build resilience.
Healthcare infrastructure	Existing systems under-equipped for climate-related health issues.	Expansion of infrastructure to support integrated care approaches.	Upgrade facilities and equipment to handle specific climate-related health challenges.
Screening and intervention	Traditional tools may not detect psychological distress caused by environmental changes.	Introduction of new tools sensitive to environmental grief, solastalgia, and eco-anxiety.	Implement new screening protocols and tools in primary care settings.
Collaborative care	Isolation from specialists in environmental and mental health fields.	Interdisciplinary teams including environmental health specialists and mental health experts.	Foster collaborations across disciplines to enhance patient care.
Holistic health approach	Current practices may not consider environmental contexts of health adequately.	Evolving practices to include environmental health in routine evaluations.	Train primary care teams to incorporate environmental health assessments into routine care.
Community resilience	Increased vulnerability of marginalized populations to climate-related mental health challenges.	Strengthened resilience through proactive mental health care and support networks.	Develop targeted outreach and support programs for vulnerable groups.
Funding and investment	Financial constraints on expanding services to include climate-related mental health care.	Advocacy for more funding and resources dedicated to mental health and climate resilience.	Secure grants and funding for long-term mental health support projects.
Long-term strategies	Lack of long-term mental health support for climate disaster survivors.	Implementation of sustainable health strategies based on WHO frameworks for disaster resilience.	Develop long-term mental health care plans that integrate climate resilience and recovery measures.

While the connection between climate change and mental health is clear, considering other factors such as genetics and personal traumas is crucial. We must also ponder whether primary care should handle these specialized issues alone. Additionally, burdening the already strained primary care sector with climate-related mental health challenges might dilute care quality. Is it worth considering a specialized avenue for such issues, separate from primary care? The variability in individual responses to environmental stressors underscores the need for fostering resilience (16). Moreover, the financial and logistical challenges of overhauling primary care to accommodate this new dimension require careful consideration.

## Immediate and long-term psychological impacts

To appreciate the gravity of the matter, one need only observe the aftermath of climate-related disasters (6). The turmoil following such events isn't merely the visible destruction, but also the invisible scars borne by survivors. Natural disasters resulting in personal loss, loss of loved ones, loss of homes, and loss of a sense of security. While the physical reconstruction following these climate-change disasters might be swift, the psychological wounds often linger, giving rise to PTSD, depression, and increased suicidal ideation (13). Furthermore, the anticipation and uncertainty surrounding future disasters contribute to chronic anxiety, fostering feelings of helplessness and pessimism among vulnerable populations.

To further complicate the landscape of mental health in the context of climate change, it is crucial to acknowledge that these impacts do not occur in a vacuum but rather exacerbate existing health inequalities. Vulnerable and marginalized populations, often burdened disproportionately by socio-economic and health disparities, face a heightened risk of adverse mental health outcomes (17). This recognition is critical for primary care practitioners, who must navigate these compounded challenges when implementing mental health interventions in an era marked by environmental distress.

In the immediate aftermath of climate-related disasters, the surge in acute stress reactions and post-traumatic stress disorder (PTSD) among affected populations is well-documented. A study by Lowe et al. (3) in the wake of Hurricane Sandy highlighted significant increases in PTSD symptoms among residents in the most affected areas. These findings underscore the urgent need for primary care practitioners to be equipped with strategies to address the immediate mental health impacts of climate-related disasters.

Beyond the immediate aftermath, the long-term psychological impacts of climate-related disasters manifest as chronic mental health conditions, including prolonged depression, anxiety disorders, and sustained PTSD. A longitudinal study by Norris et al. (7) on the long-term effects of natural disasters found that a significant portion of affected individuals exhibited persistent symptoms of depression and PTSD several years post-disaster. Moreover, a review by Hayes et al. (8) highlighted that the anticipation of future climate-related events could lead to chronic stress and anxiety, further exacerbating the long-term mental health challenges faced by communities. These studies emphasize the complex, enduring mental health repercussions of climate change, reinforcing the need for long-term mental health support strategies within primary care settings.

## Integrating climate resilience into healthcare systems

In this context, the WHO's “Operational Framework for Building Climate Resilient Health Systems” (15) and “Health Emergency and Disaster Risk Management Framework” (16) are pivotal in guiding our approaches to integrate climate resilience into primary care systems. These frameworks offer valuable insights into how health systems can anticipate, respond to, and recover from climate-related disruptions, ensuring sustained mental health support.

To address the profound impact of climate change on mental health, primary care physicians must update their training to include climate-related mental health insights. They should integrate environmental stress screenings into routine check-ups and establish connections with mental health specialists trained in ecological grief and solastalgia.

Integrating insights from environmental science, psychology, sociology, and public health into primary care can significantly enhance our approach to climate-induced mental health issues (4–6). Environmental scientists highlight the direct link between increased exposure to natural disasters and the escalation of mental health conditions, emphasizing the need for primary care practitioners to be vigilant in recognizing symptoms related to environmental stressors (11). Psychologists bring attention to the concept of eco-anxiety and solastalgia, psychological states that are becoming increasingly prevalent as individuals grapple with the rapid changes in their natural environments and the loss of their homes to climate change (18). This nuanced understanding of climate-related psychological distress is crucial for primary care providers in identifying and addressing these emerging mental health concerns.

From a sociological perspective, the disruption of social networks and community cohesion due to environmental disasters can exacerbate feelings of isolation and depression, making it imperative for primary care settings to incorporate social support mechanisms in their response to climate-related mental health challenges (12). Public health research further underscores the importance of a multidisciplinary approach, advocating for the integration of mental health services within primary care to provide a more holistic and accessible form of care for those affected by climate change (11). By adopting a cross-disciplinary perspective, primary care can evolve into a more resilient and responsive system, capable of addressing the complex interplay between climate change and mental health.

To effectively integrate the imperative of addressing climate-induced mental health issues within primary care, policy recommendations are crucial. Such programs would equip practitioners with the skills needed to address climate-related anxieties, solastalgia, and other psychological impacts of climate change (3, 6). Moreover, healthcare infrastructure must be adapted to support these initiatives, potentially requiring the expansion of mental health services within primary care settings and the establishment of interdisciplinary teams that include environmental scientists and mental health specialists (2, 4).

To support these changes, increased funding for research into the mental health effects of climate change is essential. This would enable the development of evidence-based interventions and screening tools specifically designed to detect climate-related psychological distress. Policymakers must prioritize the integration of climate change and mental health considerations into healthcare planning and resource allocation to ensure that primary care settings are adequately equipped to respond to this emerging challenge (Table 1) (6, 9).

## Stakeholder roles and policy recommendations

Ultimately, to navigate the broad implications of climate-induced mental health challenges, it is imperative to engage diverse stakeholders (19, 20). Professional associations can lead in developing guidelines and training modules for practitioners. Ministries of health and primary care networks can facilitate the integration of these guidelines into practice and ensure that sufficient resources are allocated. Individual primary care providers are crucial as they are often the first point of contact for individuals experiencing climate-related distress.

To further strengthen the support system within primary care, it is essential to direct practitioners to existing evidence-based clinical guidelines that address common mental health conditions, ensuring adherence to best practices. Concurrently, recognizing the unique challenges posed by climate-related psychoterratic syndromes, there is a critical need to develop and integrate new evidence-based interventions (21, 22). These should not only focus on psychological aspects but also consider broader ecological and societal impacts, thereby providing a comprehensive response to the multifaceted nature of these disorders. This dual approach will ensure that primary care settings are equipped to address both traditional and emerging mental health challenges effectively.

Following the directives of the WHO's “Mental Health and Climate Change: Policy Brief” (23), this paper advocates for targeted mental health strategies that address the psychological impacts of climate change. These strategies should include enhancing the capacities of primary care practitioners to manage climate-induced psychological distress, as recommended by WHO/WONCA's “Integrating Mental Health into Primary Care: A Global Perspective” (24). This guide serves as a crucial resource for implementing integrated mental health services at the primary care level, ensuring that mental health considerations are not overlooked in the face of environmental challenges.

To enact meaningful change, it is essential to delineate the responsibilities of various key stakeholders: primary care networks and agencies must implement these policies and resources at a local level, ensuring that primary care facilities are prepared to handle the increasing mental health load due to climate change. Professional associations should advocate for and develop specialized training and resources to equip healthcare providers with the necessary tools to address climate-related mental health issues (25). While, ministries of health need to create supportive policies and funding mechanisms that enable the integration of mental health considerations into the primary care settings. Furthermore, individual healthcare providers are on the frontlines, required to apply new knowledge and resources in their daily practice to detect and manage climate-related psychological distress.

To overcome these barriers, policy recommendations should focus on developing and implementing training programs tailored to the needs of primary care practitioners, enhancing funding for mental health resources, and fostering a policy environment that supports integrated care approaches (26). Leveraging facilitators involves promoting interdisciplinary collaboration between environmental scientists, mental health specialists, and primary care providers, and harnessing community engagement to build resilient healthcare systems that can withstand and adapt to the challenges posed by climate change.

## Innovative models for climate-related mental health care

To effectively incorporate climate-related mental health into primary care, innovative models must be developed. One such model could involve the integration of new screening tools specifically designed to detect signs of climate-related psychological distress. These tools would be sensitive to the nuanced ways in which environmental changes impact mental health, enabling early identification and intervention (16, 19, 25). For instance, the use of questionnaires that assess feelings of environmental grief, anxiety related to climate change, and symptoms of solastalgia could become standard practice in patient evaluations. This approach not only acknowledges the direct impacts of climate change on mental wellbeing but also validates the patient's experiences, facilitating a more empathetic and comprehensive care strategy (21, 22).

Furthermore, the primary care setting should evolve to incorporate environmental health into routine patient history taking. By asking patients about their environmental exposures, concerns, and the perceived impact of climate change on their wellbeing, healthcare providers can gain valuable insights into the environmental determinants of health. Additionally, establishing partnerships with environmental health specialists can enhance the primary care provider's ability to address these complex issues. Such collaborations could lead to interdisciplinary care teams capable of addressing both the psychological and environmental aspects of patient health, ensuring a holistic approach to care (11, 19, 26). These innovative care models, rooted in a deep understanding of the interconnectedness of environmental changes and mental health, have the potential to transform primary care into a more responsive and effective system for addressing the mental health challenges posed by climate change.

## Conclusion

The integration of primary care, mental health, and climate change has broad implications for the medical community and society at large (9). For the medical field, it signifies a paradigm shift where healthcare doesn't just respond to symptoms but also to the environmental contexts inducing them. It implies a potential surge in patients presenting with mental health issues linked to climate anxieties, necessitating a reorientation of healthcare strategies, resources, and training to cater to this emergent patient population. Beyond individual health, it also indicates a larger societal dialogue, prompting communities to struggle with collective psychological traumas induced by a shifting environment (10). The broader implications reverberate through policy, education, and community health initiatives, emphasizing the interconnectedness of our environment, wellbeing, and healthcare structures (25, 26).

To ensure a sustainable approach to mental health in the face of climate change, it is imperative to draw upon established frameworks like the WHO's “Health Emergency and Disaster Risk Management Framework” (16). This framework not only provides a blueprint for health systems to handle acute and long-term health challenges arising from climate disasters but also emphasizes the importance of integrating these strategies into primary care practices. Such integration ensures that mental health responses are swift, effective, and culturally sensitive, tailored to the needs of affected communities.

Concluding, while the challenges loom large, they also present a unique chance for reinvention. The goal is to shape a proactive primary care system, poised to address the deep-seated mental impacts of our changing climate.

## Author contributions

WJ: Conceptualization, Formal analysis, Methodology, Resources, Visualization, Writing—original draft, Writing—review & editing.
